# Ocean transit times: from basin to planetary scales

**DOI:** 10.1017/jfm.2025.11047

**Published:** 2026-01-02

**Authors:** Paola Cessi

**Affiliations:** Scripps Institution of Oceanography, University of California, San Diego, USA

**Keywords:** ocean circulation, ocean processes, dispersion

## Abstract

Lagrangian transit times on basin to planetary scales are controlled by the interplay of multiscale processes. The primary advective time scale is set by throughflow currents, such as interhemispheric western boundary currents. Dispersion by mesoscale eddies introduces fluctuations that erase memory and enhance dispersion, widening the transit-time distribution. The tortuous paths of Lagrangian parcels, particularly within ocean gyres, significantly enhance dispersion beyond the levels attributed to mesoscale eddies alone. Additionally, trapping by ocean gyres leads to multimodal distributions of Lagrangian transit times. These processes are illustrated in three complementary contexts: eddy-permitting ocean state estimates, simplified spatially extended three-dimensional flows and diffusively coupled two-dimensional pipe models.

## Graphical abstract

**Figure:**
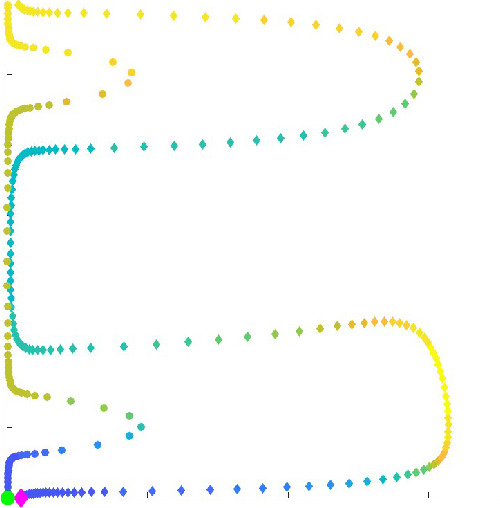

## 1. Introduction

Information about the time scales and pathways between regions in the ocean can be obtained by either tracking the advection-diffusion of tracers or following Lagrangian trajectories. Both methods are used in observational campaigns, using transient tracers (Fine 2011) or Lagrangian floats (Bower *et al.* 2019). The observational studies have been complemented by numerical studies, where tracers (Haine & Hall 2002; Holzer & Primeau 2007; DeVries & Primeau 2011; Shah *et al.* 2017) or parcels are advected and diffused using synthetic velocity fields produced by ocean models (Döös 1995; Blanke & Raynaud 1997; Speich, Blanke & Cai 2007; Rousselet, Cessi & Forget 2021).

The statistics of tracers and Lagrangian parcels, represented by either tracer concentrations or probability distributions of the parcel’s density in space and time, are equivalent as long as the boundary and initial conditions are consistent (Shah *et al.* 2017). The Lagrangian method allows efficient computations in the limit where the diffusion time is much longer than the advection time, a regime that is difficult to achieve with Eulerian methods. In the following, we focus on the first passage time statistics derived from Lagrangian analysis. First passage time is defined as the time when a stochastic walker or a diffusing parcel first reaches a given site starting from another given site (Redner 2001).

In the oceanic context, the dispersion by mesoscale eddies is a well-studied feature in Lagrangian analysis, which can be quantified in terms of the diffusivity, 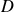 , (Davis 1991*a*, *b*). The effect of eddies might be considered to be analogous to the diffusion induced by stochastic noise in probability space in the Fokker–Planck (or Kolmogorov) equation describing the probability of parcels being at a point in space at a given time, when advected by a random velocity. One implicit assumption is that `eddies’ have smaller scales, in space or time, than the `mean’ component of the velocity. Additional assumptions are that eddies transport tracers down the mean gradient, at least in some direction, e.g. along isopycnals.

Oceanic flows on the planetary scale have multiscale motions including throughflow currents that are typically confined to a boundary, but span the full meridional extent of basins across two hemispheres, e.g. the western boundary currents associated with the Atlantic meridional overturning circulation (AMOC). On the basin scales are large-scale gyres, which also include narrow boundary currents on the same scale of the throughflow and of mesoscale eddies. Gyres, in particular, trap trajectories into spiralling three-dimensional (3-D) motions (Rousselet *et al.* 2021; Rousselet & Cessi 2022; Vecchioni *et al.* 2023). Mesoscale eddies diffuse tracers efficiently along isopycnals, delaying the progression of parcels and tracers inside boundary currents. The net effect of gyres and eddies is to broaden the transit-time distributions of tracers and parcels travelling meridionally. In this work we focus on the upper ocean circulation, i.e. the flow in the top kilometre of the water column, which is the best-observed region of the ocean. We attempt to tease apart the contribution of throughflows, gyres and eddies to the transit times between sites separated in latitude.

Several works successfully fit the first passage time distribution (FPTD) of Eulerian tracers or Lagrangian parcels in the ocean as an inverse Gaussian distribution of the form (Hall, Haine & Waugh 2002; Waugh, Hall & Haine 2003; Peacock & Maltrud 2006; Chouksey *et al.* 2022)
(1.1)

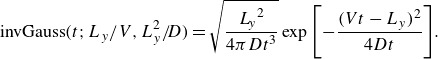

The inverse Gaussian (1.1) arises in the probability distribution of first passage times for parcels, in one dimension, starting at 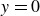 and arriving at 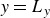 subject to a uniform drift, 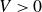 , and Brownian motion with zero mean and variance given by 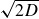 (Cox & Miller 1965, Chapter 5.7). The form (1.1) clarifies that the parameters of the inverse Gaussian can be interpreted in terms of advective, 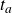 , and diffusive, 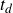 , times associated with the underlying Fokker–Planck equation. The two parameters are given by
(1.2)

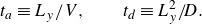

In (1.1) the relevant diffusive time is along the direction of the constant velocity, 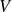 , while in higher dimensions, diffusion across the mean velocity is another relevant parameter.

The inverse Gaussian distribution is characterised by a small but finite probability at short times. This is because rare but large fluctuations of the stochastic component can take parcels from the starting point to the arrival target in one random step. Equivalently, for finite but non-zero diffusivity, an exponentially small but finite tracer concentration is diffused immediately along a line, before the advective propagation. At large times, the inverse Gaussian distribution decays exponentially, because the advection can efficiently flush out parcels (or tracers), despite the diffusive backscatter.

An alternative to the inverse Gaussian distribution is the (one-sided) Levy distribution, given by
(1.3)

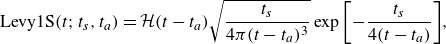

where 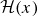 is the Heaviside function. As for the inverse Gaussian, the Levy distribution contains two parameters: the shift parameter (sometimes called `location’ parameter), 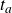 , represents a threshold time that needs to be overcome before the target site can ever be reached; the so called `scale’ parameter, 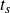 , determines the position of the distribution maximum relative to 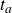 (Nolan 2020). Unlike the inverse Gaussian, Levy1S vanishes for short times, 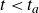 . Contrary to the inverse Gaussian, the Levy distribution decays slowly for large times, as 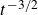 . This heavy tail causes all the moments larger than zero to diverge. We show in § 5 that the distribution (1.3) is appropriate for first passage times of parcels in two or three dimensions, advected by a narrow throughflow current with minimum advection time 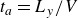 , and subject to noise/diffusion across the throughflow current, into a reservoir much wider than the width of the throughflow current. Because of the diffusive hold-up into the reservoir, parcels’ progression is slowed down and the tail of the FPTD is heavy.

In § 2 it is shown that the two distributions, invGauss and Levy1S, arise in two separate regions of the ocean, the South Atlantic Ocean and the Western Mediterranean Sea, where different processes control the dynamics.

To our knowledge, the Levy1S distribution has not been discussed in the oceanographic context. The trapping by excursions in regions of temporary retention, such as gyres, has been studied in the context of transport of tracers (Young 1988) and porous media (Hidalgo, Neuweiler & Dentz 2021). Here, we revisit the problem in the oceanographic context, adding together throughflow boundary currents on the planetary scale, basin-scale gyres and stochastic noise (parametrising mesoscale eddies) in the advection of Lagrangian parcels, i.e. the main elements of the circulation in the top kilometre of the ocean. In § 4 it is shown that the distribution invGauss is appropriate for first passage times when, in the presence of eddies, the western boundary currents associated with gyres overcome the throughflow boundary current. In this case, the effective diffusivity in the direction of the throughflow boundary current is enhanced above the value estimated from eddy dispersion alone. Conversely, the Levy1S distribution dominates the fast time component of the transit-time distribution when the throughflow boundary currents velocity overcomes the opposing western boundary current associated with the gyral transport (also in the presence of eddies).

This study also shows that large-scale gyres lead to multimodal distributions, in addition to holding up parcels for long periods. This implies that multimodal distributions are not necessarily due to different origins of parcels or water masses, but rather to different routes of parcels with contiguous origins.

The retarding and dispersive effects of gyres and eddies is illustrated using a hierarchy of models of decreasing complexity, with the common process of Lagrangian parcels advection. The most complex models are eddy-permitting state estimates used to advect virtual parcels in basin-scale regions that accommodate three types of flows: (i) currents that flow through the open boundaries of the domain; (ii) permanent gyres on scales comparable to the domain, which recirculate parcels in 3-D spiralling motions; (iii) transient mesoscale eddies on scales much smaller than the region of interest. All three types of flows contribute to the trajectories connecting the open boundaries of the region of interest. The FPTDs in these complex 3-D, time-dependent flows and in complicated geometrical domains are discussed in § 2: they motivate the formulation and analysis of simplified models.

The intermediate complexity model uses the 3-D steady velocity solution of the linear barotropic momentum equations in a semi-enclosed domain forced by a simplified surface wind stress with three gyres plus a north–south throughflow boundary current flowing across the open boundaries. The throughflow current is a crude representation of the interhemispheric throughflow boundary current associated with the upper branch of the AMOC. The formulation of this velocity field is detailed in § 3. The total velocity field advects Lagrangian parcels, which are also subject to stochastic Brownian motion to account for time-dependent flows on smaller scales. This framework allows us to explore different regimes of relative importance of gyres versus thoughflow currents, and the role of eddies (noise) in enabling the gyral dispersive effect. The results of the stochastic Lagrangian advection is discussed in § 4.

The simplest models consider constant flows through pipes that are diffusely coupled in two dimensions. This simplification allows us to derive the explicit form of the FPTD as a function of the parameters of the model. The pipe models are discussed in §§ 5 and 6.

## 2. Motivation: Lagrangian parcels advected by eddy-permitting state estimates

In this section we discuss the FPTD resulting from Lagrangian analysis of virtual parcels advected with eddy-permitting time varying 3-D velocity fields from two state estimates: the Southern Ocean state estimate (SOSE, Swierczek *et al.* 2021) and the Mediterranean Sea reanalysis (MED REA) (Simoncelli *et al.* 2014). Both state estimates assimilate *in-situ* vertical profiles of temperature and salinity from ship-based instruments and autonomous Argo floats, in addition to sea-surface height and sea-surface temperature from satellites. The data are assimilated into ocean–sea-ice models with closed budgets of mass, momentum, temperature, salinity and sea ice, by adjusting initial conditions, and also boundary conditions for SOSE, to minimise the observations–model misfit. Both eddy-permitting models advect tracers and momentum with no explicit horizontal diffusion or viscosity: there is explicit biharmonic horizontal diffusion and viscosity with coefficients 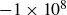 m 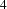 s^−1^ for SOSE and 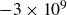 m 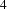 s^−1^ to remove the accumulation of gradients at the grid scale. But the unavoidable and unknown implicit diffusion due to the finite difference advection schemes probably dominates on scales larger than the grid size. The background vertical diffusion is kept to a minimum ( 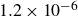 m 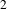 s^−1^ in MED REA and 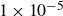 m 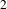 s^−1^ in SOSE). Various dynamically controlled enhancements of vertical mixing are included to avoid unstable stratification.

The details of the SOSE Lagrangian analysis are given in Rousselet, Cessi & Mazloff (2023). Briefly, about eighty thousand parcels are seeded in the Atlantic sector near the equator in the upper branch of the AMOC ( 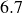 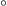 S above 1200 m), and then followed backwards in time to either the Drake Passage (66 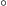 W at any depth) or to a section between the tip of South Africa and Antarctica (22 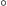 E at any depth). The SOSE velocity field is resolved at 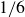 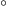 in the horizontal and with 52 vertical levels. Because 79 % of parcels originate from the 22 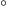 E section, we focus on the FPTD for this group, which has the best statistics.

The details of the MED REA Lagrangian analysis are given in Vecchioni *et al.* (2023). Briefly, about four million parcels are seeded at the Strait of Gibraltar ( 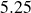 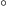 W at any depth) and then followed backwards in time in the Western Mediterranean Sea to either the Gulf of Lions, or the Tyrrhenian Sea (both at 42.31 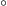 N at any depth), or the Strait of Sicily (36.94 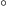 N at any depth). The MED REA velocity field is resolved at 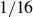 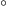 in the horizontal and with 72 vertical levels. Because 86 % of parcels originate from the Gulf of Lions section, we focus on the FPTD for this group, which resolves even the tail of the distribution.

In both the SOSE and MED REA, each parcel is assigned a transport at release, by dividing the total transport across the initial grid face by the number of parcels released at that face. Because the flow is incompressible, each parcel’s initial transport is conserved following its trajectory.

**Figure 1.**
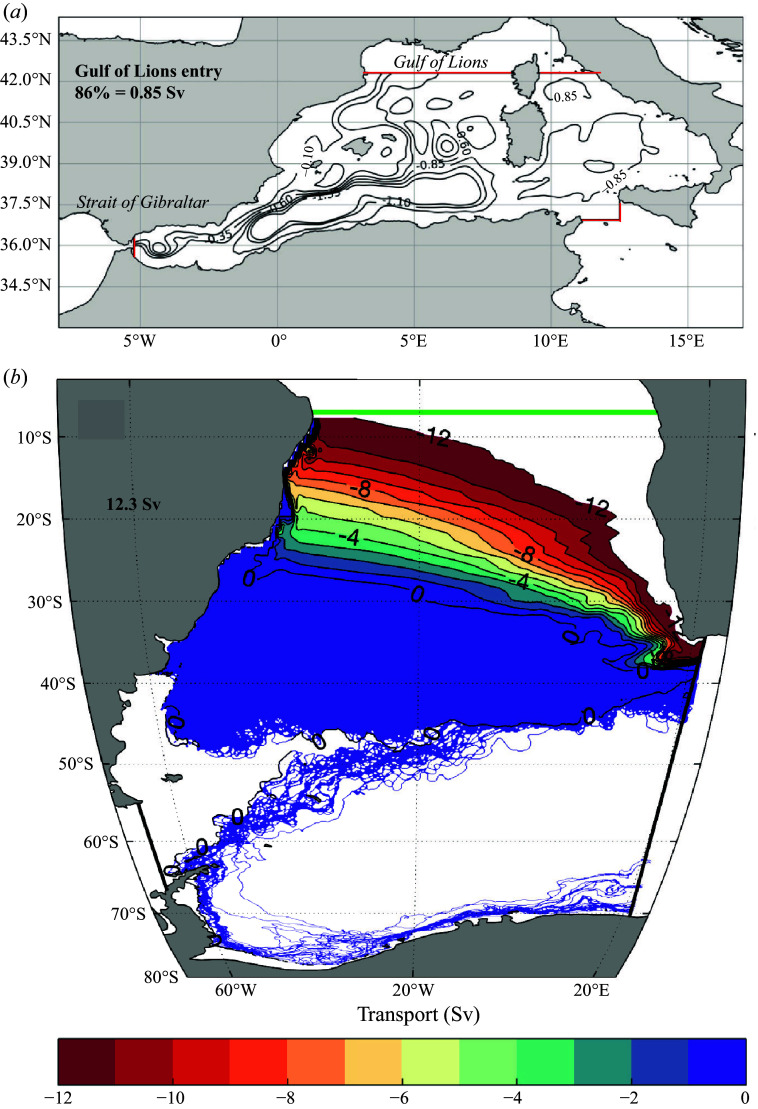
Ensemble-averaged and vertically integrated horizontal streamfunctions of Lagrangian parcels connecting two sections (displayed forward in time). (*a*) Paths from the Gulf of Lions (42.31 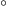 N section in red) to the Strait of Gibraltar (5.25 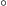 W section in red) as represented in MED REA in units of Sv (black contours, but contours greater than 1.35 Sv are omitted). (*b*) Paths from a section at 22 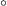 E (connecting the tip of South Africa to Antarctica, thick line in black) to the equatorial region in the South Atlantic (6.7 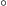 S section denoted by the green thick line) as represented in SOSE in units of Sverdrups ( 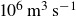 , Sv). Because there are only a few trajectories that take the retroflecting paths south of 50 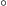 S, the streamfunction shows the individual trajectories.

Figure 1 shows the ensemble-averaged vertically integrated streamfunctions as a function of latitude and longitude for the two types of trajectories considered: from the 22 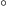 E section to the equatorial region in the South Atlantic as represented in the SOSE (panel *b*); from the Strait of Gibraltar to the Gulf of Lions as represented in the MED REA (panel *a*). The ensemble-averaged streamfunction is constructed by summing the components of the horizontal transport carried by each parcel over all depths and occurrences in latitude–longitude bins and then summing the resulting east–west transport in latitude or the north–south transport in longitude (Blanke *et al.* 1999). This procedure produces a two-dimensional streamfunction in the latitude–longitude plane. Thus, some 3-D spiralling motions are shown as closed contours in the Mediterranean Sea, even though each parcel path is open in three dimensions, connecting the entry and exit sections.

**Figure 2.**
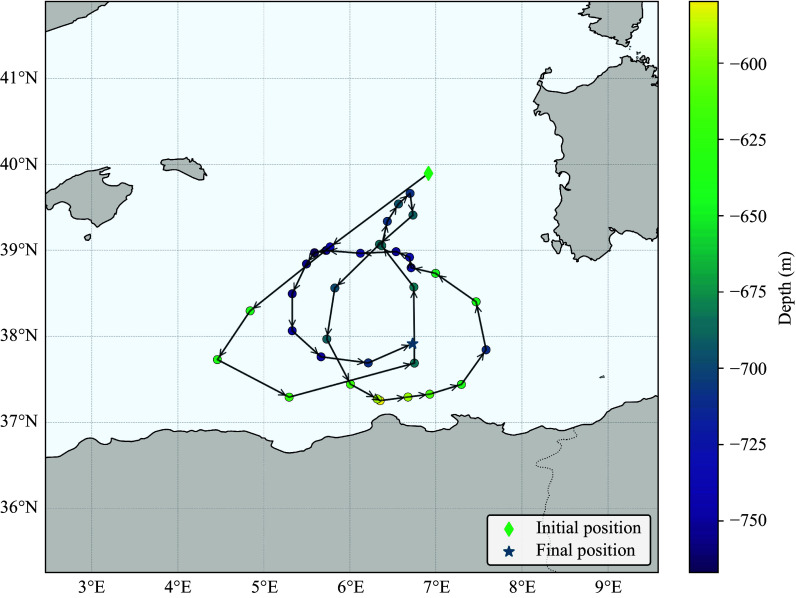
Portion of a trajectory in the south Western Mediterranean, 280 days long, sampled every eight days. The arrows indicate the direction between two contiguous samples, the colour of the filled symbols indicates depth according to the colourbar on the right-hand side. The initial position is marked by a filled diamond and the final position by a filled star. Intermediate positions are marked by filled circles.

The two streamfunctions are qualitatively different in that the typical paths of the AMOC in the South Atlantic are direct and squeezed into a western boundary current along the South American coast (figure 1 *b*). Conversely, the Mediterranean paths are tortuous, punctuated by spiralling recirculations around several permanent gyres (figure 1 *a*). An example of the portion of a single trajectory shown in figure 2 illustrates the 3-D spiralling motion on the sub-basin-scale gyres, larger than the 50 km diameter typical of mesoscale eddies in the Western Mediterranean.

**Figure 3.**
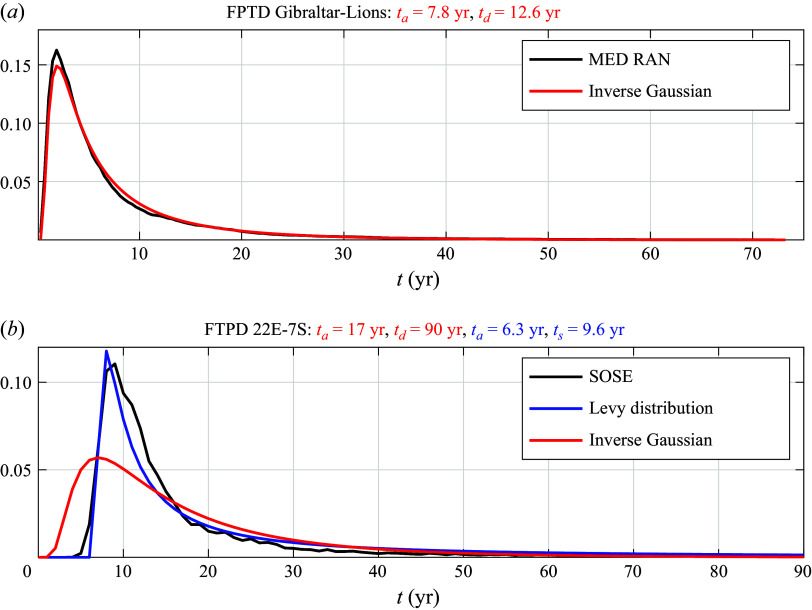
Lagrangian histograms of first-passage transit times weighted by the parcels transport for the Gulf of Lions to Strait of Gibraltar trajectories in MED-RAN (panel *a*, black line) and for the 22 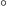 E section south of the tip of Africa to the equator in the Atlantic sector in SOSE (panel *b*, black line). The Lagrangian histograms are compared with inverse Gaussians of the form (1.1), with the two parameters 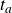 and 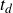 fitted using the maximum likelihood estimator (MLE) as in (2.1) (red lines) or with a Levy distribution as in (1.3) (blue line). The parameters of the distributions, defined in the text, are reported in the titles of each panel: red numbers are for the inverse Gaussian and blue numbers are for the Levy distribution. The two parameters of the Levy distribution are obtained by the least-square fit.

**Figure 4.**
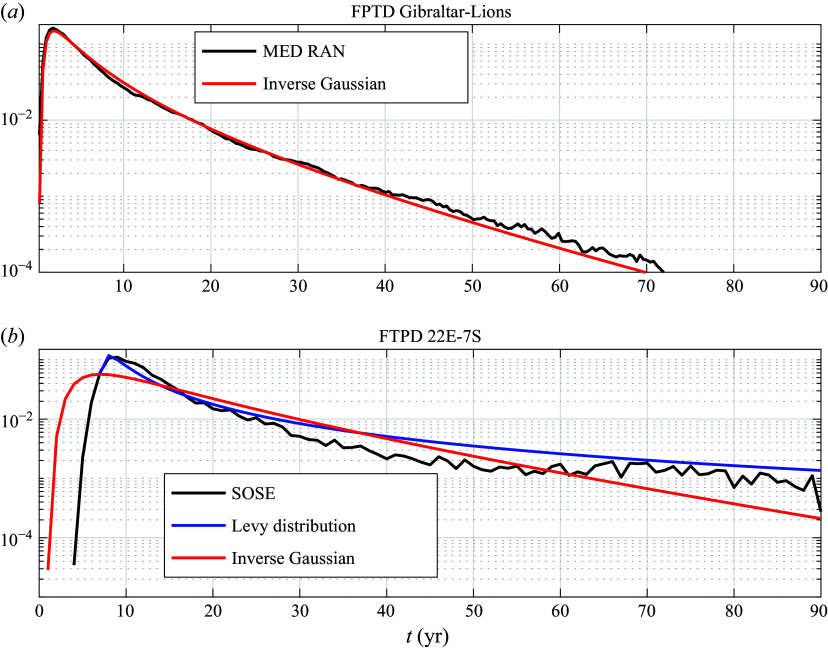
Same as figure 3, except in logarithmic scales.

The corresponding Lagrangian (i.e. transport-weighted) FPTDs are displayed in figures 3 and 4 for the SOSE (bottom) and MED REA (top). Both distributions are compared with inverse Gaussians by fitting the first two moments (Hall *et al.* 2002; Waugh *et al.* 2003; Peacock & Maltrud 2006; Chouksey *et al.* 2022). Specifically, the first and second moment are calculated with the transport-weighted maximum likelihood estimator (Shao *et al.* 2016), i.e.
(2.1)

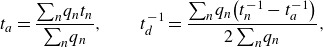

where 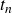 and 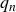 are the arrival time and transport of each parcel, respectively. For the MED REA parcels, the inverse Gaussian works well with 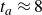 years and 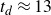 years. In this case, the effective dispersion time is comparable to the advection time across the domain.

In contrast, the fit to an inverse Gaussian (red curve in figures 3 *b* and 4 *b*) for SOSE is unsatisfactory. A better fit is provided by the Levy distribution (1.3) (blue curve in figures 3 *b* and 4 *b*). The important differences between the two theoretical distributions, most apparent in figure 4 *b*, is the lack of an exponential tail in (1.3) and the shift of the distribution by 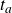 . We ascribe the difference in the MED REA versus SOSE FPTDs not to different statistics of mesoscale eddies in the two regions, but to the trapping of trajectories by permanent gyres in the MED REA, which are absent in the SOSE velocity field for the domain considered. In § 5 we show that the Levy distribution represents the FPTD of parcels advected by a narrow current with diffusion across the current into a region much wider than the current’s width, as appropriate for the configuration in SOSE. Conversely, in § 4 the effect of trapping by the 3-D spiralling motion associated with permanent gyres is illustrated considering stochastic processes advected by a 3-D velocity of intermediate complexity plus noise. The details of the 3-D velocity field are given in § 3.

## 3. A 3-D steady velocity field with ocean gyres and a boundary current throughflow

To understand the interplay of permanent gyres and throughflow boundary currents on Lagrangian (or tracers) transit-time distributions, we construct a 3-D steady velocity field that includes both processes. The velocity field is the solution of the linearised, steady, primitive equations for a barotropic fluid, driven by wind stress and damped by bottom friction. We then add to this steady 3-D velocity field stochastic fluctuations to model the effects of random eddies. The resulting velocity field contains the multiscale processes that advect Lagrangian parcels in more complex contexts, such as the SOSE and MED-RAN. Each process can be studied in isolation or in different combinations to understand the resulting FPTDs.

### 3.1. Velocities in a barotropic layer

To illustrate the kinematics of Lagrangian trajectories advected by oceanic large-scale flows, we consider the simplest model of wind-driven gyres.

The steady, linear dynamics of a single layer of constant density, 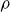 , and constant depth, 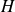 , is governed by 
(3.1)

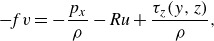



(3.2)

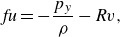



(3.3)

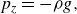



(3.4)

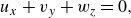




where 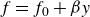 , 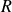 and 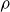 are constant. The boundary conditions are that the normal velocity vanishes on the top, bottom, eastern and western boundaries, while the northern and southern boundaries are open to allow for a throughflow, 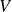 , i.e. 
(3.5)

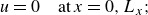



(3.6)

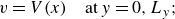



(3.7)

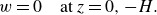




The wind-stress forcing, 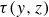 , is applied as a body force in the 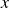 direction, defined in (3.12), and decays exponentially away from 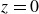 , representing the stress due to the wind forcing localised near the surface, while omitting the details of the Ekman spiral.

In the case of wind stress in the zonal direction only and independent of 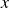 , the approximate solution for the velocity is easily derived in the limit 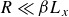 , and we can write the velocity field as (Rousselet & Cessi 2022)
(3.8)

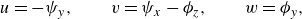

where 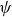 is the horizontal streamfunction, given by
(3.9)

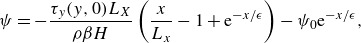

where
(3.10)

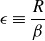

is the width of the western boundary current. The term proportional to 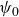 is an exact homogeneous solution of the governing equations, as long as the throughflow velocity, 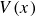 at 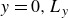 , has the appropriate exponential form, i.e. 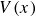 is proportional to the last term on the right-hand side of (3.9). This boundary current throughflow is a representation of the upper branch of the mid-depth overturning circulation. The strength of the gyres is controlled by the term proportional to 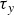 in (3.9).

The Ekman transport is associated with an overturning streamfunction, 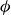 , given by
(3.11)

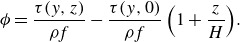




Because of the Ekman flow near the surface and its geostrophically balanced return below the Ekman layer, 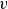 has a horizontally divergent component, 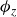 , which is associated with a vertical velocity. While the horizontal streamfunction is proportional to the wind-stress curl, the meridional overturning streamfunction is proportional to the wind stress. In this way, horizontal gyres are coupled at every depth by the Ekman transport and its return. Because 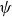 and 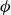 are approximately in quadrature in the latitudinal direction, regardless of where a parcel starts its journey, it will eventually sample all gyres and move in 3-D spiralling motions, while drifting through the domain because of the boundary current forced at the meridional boundaries, 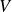 , proportional to 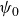 .

The effect of permanent gyres is illustrated by choosing a wind stress of the form
(3.12)

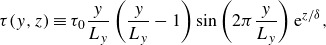

which vanishes and has zero derivative at 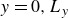 . The throughflow velocity at the boundaries 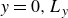 is given by 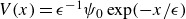 , where 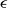 is defined in (3.10).

## 4. Lagrangian first passage times advected by 3-D flow and stochastic noise

To illustrate the trapping role of gyres, we follow Lagrangian trajectories advected by the 3-D incompressible velocity in (3.8), (3.9) and (3.11), with an additive stochastic component proportional to 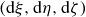 . In other words we solve the discrete equations
(4.1)

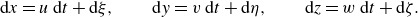

The stochastic component 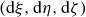 is picked randomly from a normal distribution with zero mean and standard deviation 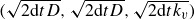 . Additionally, by enforcing reflection of parcels on the east and west boundaries, every trajectory bounces off the solid walls. However, diffusive flux, in addition to the prescribed advection, is allowed on the entry and exit boundaries at 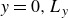 .

**Table 1.** Reference values of the parameters used in the barotropic model and in the Lagrangian trajectory calculations. The additional parameter 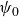 is varied throughout the Lagrangian calculations.

Parameter	Value	Notes
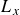	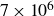 m	Basin width
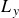	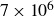 m	Basin length
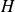	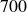 m	Basin depth
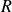	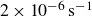	Bottom drag
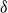	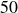 m	Ekman layer depth
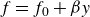		Total Coriolis parameter
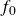	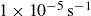	Coriolis parameter at 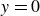
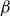	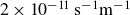	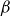 parameter
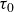	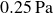	Wind-stress amplitude
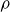	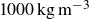	Constant density
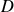	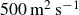	Horizontal diffusion
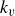	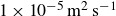	Vertical diffusion
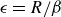	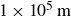	Width of western boundary layer

**Figure 5.**
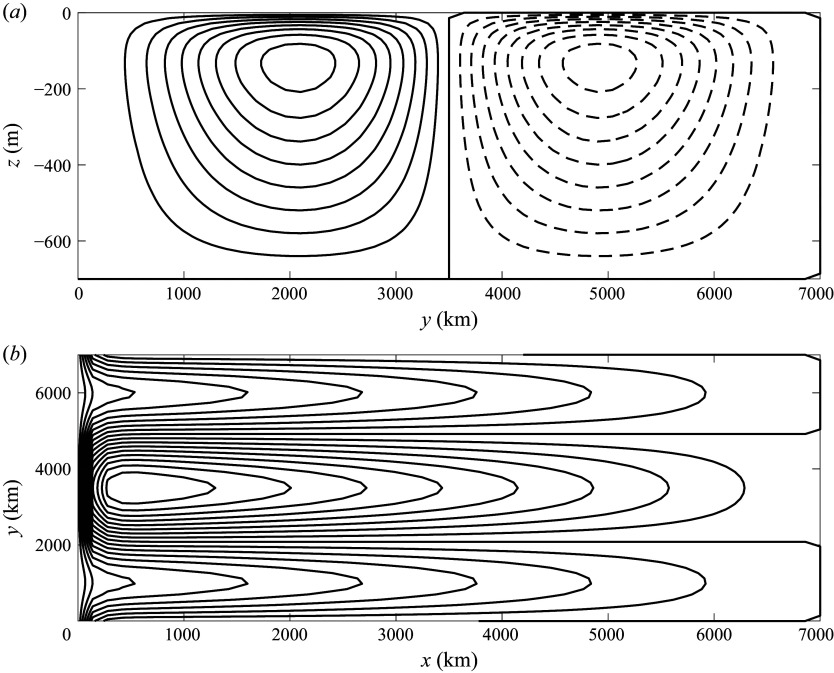
Ekman overturning streamfunction, 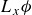 , as a function of latitude and depth (panel *a* – contour interval 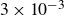 Sv, negative values are dashed). Vertically integrated horizontal streamfunction, 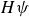 , as a function of longitude and latitude (panel *b* – contour interval 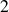 Sv, zero value at 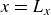 ). The expressions for 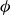 and 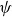 are given in (3.11) and (3.9), respectively. The parameter values are given in table 1.

Figure 5 shows 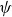 and 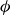 resulting from the wind stress in (3.12). The parameter values used are listed in table 1, and the net north–south transport, 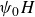 , is 18 Sv (1 Sverdrup – Sv = 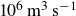 ). The maximum northward western boundary current transport associated with the gyres only (i.e. without the throughflow transport proportional to 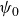 ) is also 18 Sv, while the maximum southward western boundary current transport is 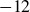 Sv (gyres only). In this example, the total transport in the western boundary current is everywhere northward. However, for 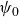 less than a critical value that controls the strength of the gyres, the total meridional velocity on the western boundary would change sign, i.e. it would be southward in the tropical and subpolar latitudes. This threshold, whose parameter dependence is derived in § 4.2, has implications for the advective paths across the meridional length of the domain.

As in the Lagrangian analysis using state estimates in § 2, the first passage time is defined as the first time a parcel starting at 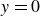 crosses 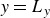 , at any longitude, 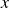 , or depth, 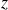 . Parcels are initialised at 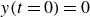 uniformly in 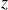 . The initial release for the 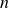 th parcel along 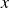 is according to 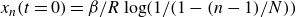 ( 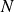 is the number of parcels per unit depth), to ensure constant meridional transport among releases along the 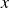 direction. This flux-weighted strategy ensures the equivalence of Lagrangian parcels and tracer release (Yang & Cessi 2024, Appendix A).

Because the steady velocity associated with the gyral circulation is three dimensional, in general the deterministic system associated with (4.1) is chaotic (Dombre *et al.* 1986). Without stochastic noise, the trajectories are sensitive to initial conditions and generically fill the whole volume of the domain, although we cannot exclude small islands of periodicity (Rousselet & Cessi 2022). Thus, trajectories entering the gyres will generically sample the whole domain, distributing the throughflow velocity associated with 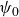 over a wide swath of longitudes and depths, even without the stochastic noise component.

In the following we focus on the parameter values given in table 1. The Lagrangian trajectories can be examined in various limits.

### 4.1. Advection by boundary current plus noise, no gyres

In the absence of gyral flow, i.e. 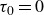 , the flow reduces to 
(4.2)

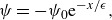



(4.3)

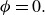




In this case, we have Brownian motion with an 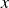 -dependent drift in the 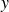 direction confined near the boundary at 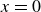 , constant in 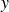 and 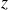 . It is tempting to neglect the diffusion by stochastic noise and estimate the FPTD considering the statistics of parcels with advection only. The resulting FPTD is derived in Appendix A and is given by
(4.4)

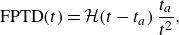

where 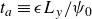 . Note the 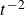 tail compared with the 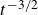 tail for the Levy1S distribution (1.3): the latter has a `heavier’ tail because diffusion outside the western boundary layer traps the parcels for long times, and thus delays their meridional progress.

**Figure 6.**
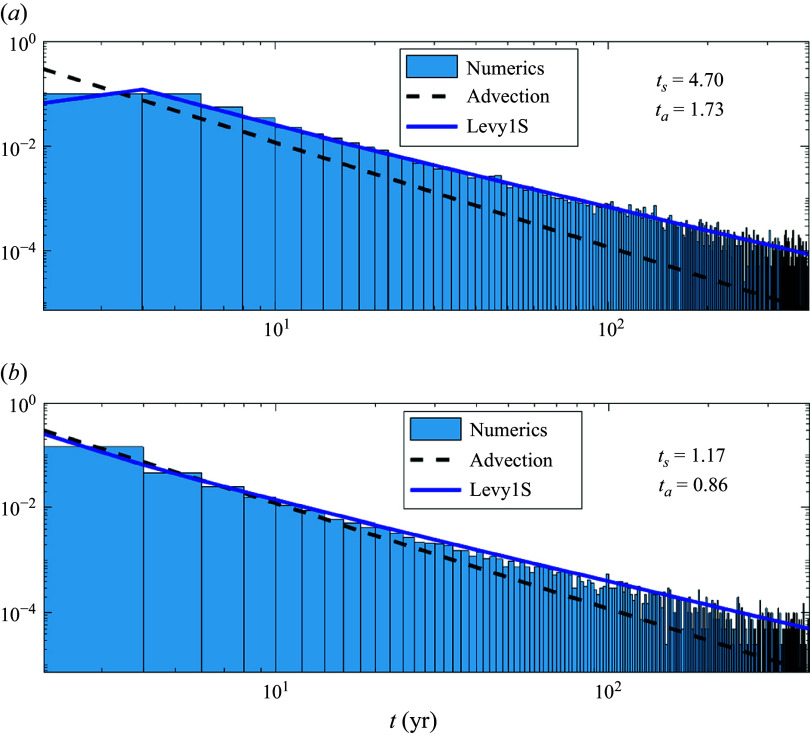
First passage time distributions (normalised to unity area integral) for the Lagrangian trajectories of (4.1), with the velocity field given in (3.9), for the parameter values in table 1, except 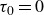 , e.g. there are no gyres (light blue histograms with 100 bins). Results are shown for (*a*) 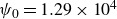 m 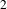 s^−1^ and (*b*) 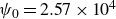 m 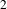 s^−1^. Also shown are the theoretical predictions (5.11) (blue lines) and (4.4) (black dashed lines), using 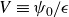 , i.e. the maximum value of the boundary current velocity. The value of the parameters of the Levy1S distribution, 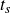 and 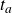 in years, are given in the figure.

Figure 6 shows the FPTDs for direct numerical solutions of (4.1) for two values of 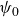 (light blue histograms), with all the other parameter values given in table 1. The FPTD (4.4), shown as a dashed black line, is not a good approximation for the tail of the distribution. In contrast, the Levy distribution (1.3) (blue line) is an excellent fit, with 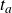 and 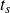 given by
(4.5)

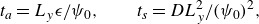

using the values tabulated in table 1.The model problem developed in § 5, with a constant pipe flow diffusively leaking into a quiescent region is an appropriate idealisation for this configuration, which allows us to determine the dependence of 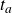 and 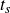 on the parameter values. In both examples shown in figure 6, 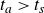 , diffusive effects cannot be neglected and the purely advective regime is not a good fit.

### 4.2. Gyres plus throughflow, no noise

**Figure 7.**
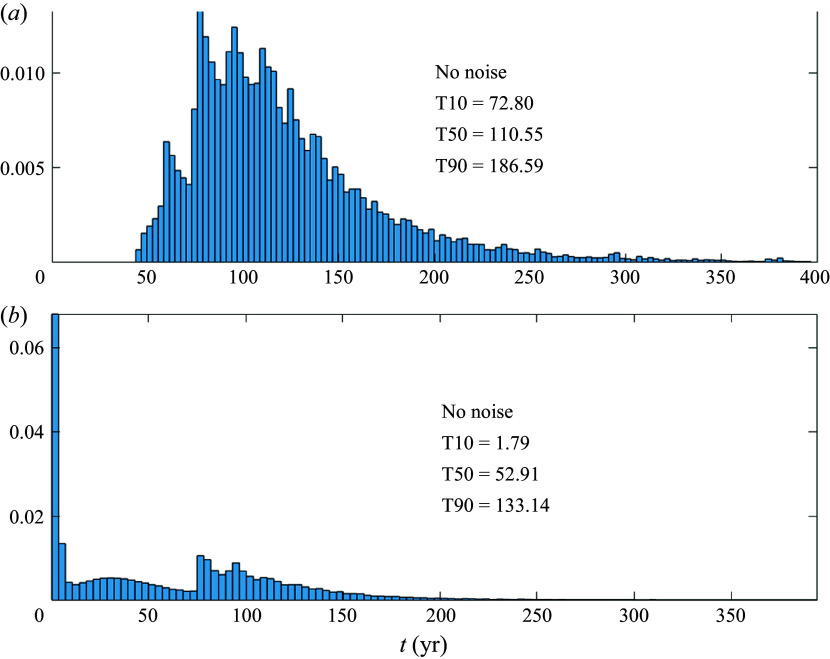
First passage time distributions (normalised to unity area integral) for the Lagrangian trajectories of (4.1), with the velocity field given in (3.9) and (3.11), for the parameter values in table 1, except 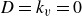 , e.g. there is no stochastic noise. Results are shown for (*a*) 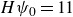 Sv, which corresponds to 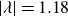 ; (*b*) 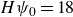 Sv, which corresponds to 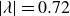 . Here T10, T50 and T90 indicate the 10-percentile, 50-percentile (median) and 90-percentile transit times of the distributions (in years).

Another interesting limit is when there are gyres plus a throughflow boundary current, but there is no stochastic noise, i.e. 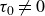 , and 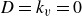 . The corresponding FPTD is shown in figure 7. The distribution is multimodal, with a slowly decaying tail. There is a large peak near 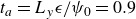 years in the case of the stronger boundary current (figure 7 *b*), which is absent for the weaker boundary current (figure 7 *a*). The disappearance of the peak at short transit times is controlled by the parameter
(4.6)

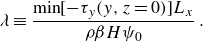

**Figure 8.**
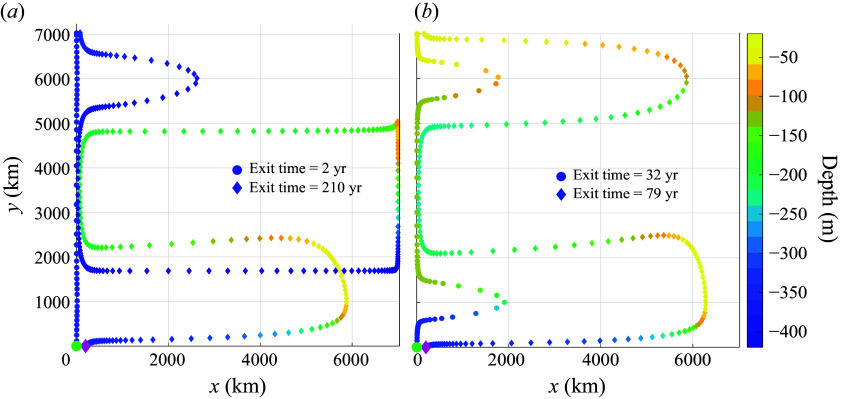
Pairs of Lagrangian trajectories all starting at 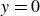 , 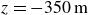 for different 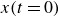 . The initial positions are marked by a green dot at 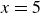 km and a magenta diamond at 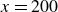 km, and all the other parameters are as in table 1, except that 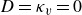 . Results are shown for (*a*) 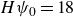 Sv, which corresponds to 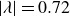 ; (*b*) 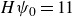 Sv, which corresponds to 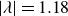 . The exit times at 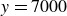 km are given in the textbox. The depth of the parcel is colour-coded according to the colourbar to the right of the panels.

When 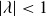 , the velocity at the western boundary is positive at every latitude. In the absence of noise, this guarantees some direct paths from 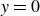 to 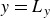 not held up by the gyres. This direct path is responsible for the peak at transit times 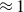 year in figure 7 *b*. Conversely, when 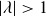 , every path from 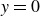 to 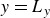 is held up in the gyres, substantially lengthening the minimum transit time, as seen in figure 7 *a*. For the parameter values in table 1, the critical value is 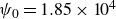 m 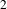 s^−1^. In both regimes the tail of the distributions does not become exponential and decays with power laws (not shown). The qualitative difference in the trajectories depending on 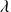 is illustrated by examples in the two regimes in figure 8. For 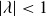 (figure 8 *a*), parcels starting very close to the western boundary at 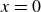 flow straight through the domain, without excursions into the gyres, with short transit times (filled circles in figure 8 *a*). Parcels starting further east spiral around gyres before exiting, leading to long transit times (filled diamonds in figure 8 *a*). For 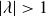 (figure 8 *b*), every trajectory experiences tortuous 3-D excursions into the gyres, leading to long transit times even for parcels starting very close to 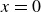 (e.g. filled circles in figure 8 *b*).

Multimodal distributions in first passage times for Lagrangian parcels have been documented in numerical ocean models of the global circulation and interpreted as arrivals of different water masses or from well-separated locations (Peacock & Maltrud 2006; Chouksey *et al.* 2022). Here the multimodality occurs because of the different paths taken by parcels as they avoid or get trapped into the gyres, while all starting nearby, all concentrated in the western boundary current. Once in the gyres, escapes occur in spiralling motions whose vertical component is associated with the overturning streamfunction, 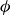 , and thus, quite slow.

**Figure 9.**
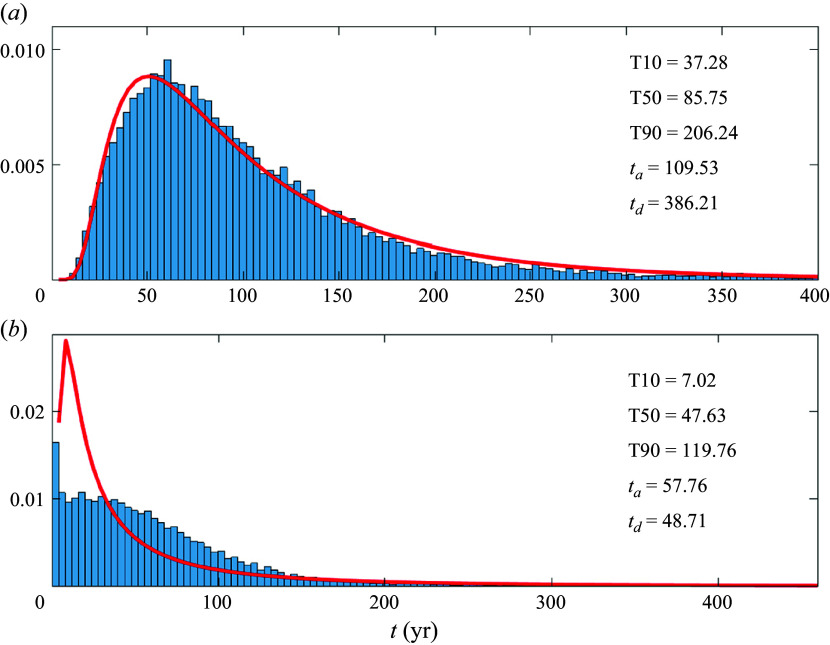
First passage time distributions (normalised to unity area integral) for the Lagrangian trajectories of (4.1), with the velocity field given in (3.9) and (3.11). All parameter values are given in table 1. Results are shown for (*a*) 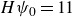 Sv, which corresponds to 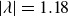 ; (*b*) 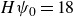 Sv, which corresponds to 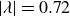 . The red curves show inverse Gaussians in (1.1) with parameters fitted by (2.1), given by 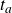 and 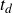 . Here T10, T50 and T90 indicate the 10-percentile, 50-percentile (median) and 90-percentile transit times of the distributions (in years).

### 4.3. Gyres, boundary currents and noise

When stochastic noise is added to the gyral and throughflow velocities, using the reference values in table 1, the qualitative nature of the FPTD depends on the parameter values. For 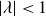 , the deterministic velocity at the western boundary is positive at every latitude: a peak at transit times of the order of the fastest advective time is thus present even with noise (figure 9 *b*). When 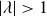 , all advective paths have to go through the gyral circulation. In this regime, the one-dimensional inverse Gaussian distribution is a good fit (figure 9 *a*, red curve). Gyres enhance the dispersion in the meridional direction beyond the diffusivity values typically expected for mesoscale eddies. In the example in figure 9 *a* the stochastic noise variance corresponds to a diffusivity 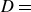 500 m 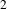 s^−1^ (cf. table 1), but the fitted 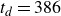 years corresponds to an effective diffusivity of 4000 m 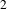 s^−1^. Note that the dispersive enhancement by gyres is mediated by the explicit diffusivity 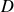 (cf. figures 6 *a*, 7 *a* and 9 *a*). In the regime 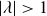 , gyres homogenise the effective throughflow meridional velocity so that the inverse Gaussian distribution, which assumes a constant throughflow, is a good approximation to the FPTD.

Trapping in the gyres delays the transit times relatively to the advective time associated with the throughflow and, with explicit diffusion, gyres collectively enhance the dispersion. For example, in figure 9(*a*) the advective and diffusive time scales of the inverse Gaussian are comparable, even though the Peclet number of the flow, as measured by 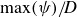 , is much greater than one (in this case 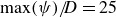 ).

**Figure 10.**
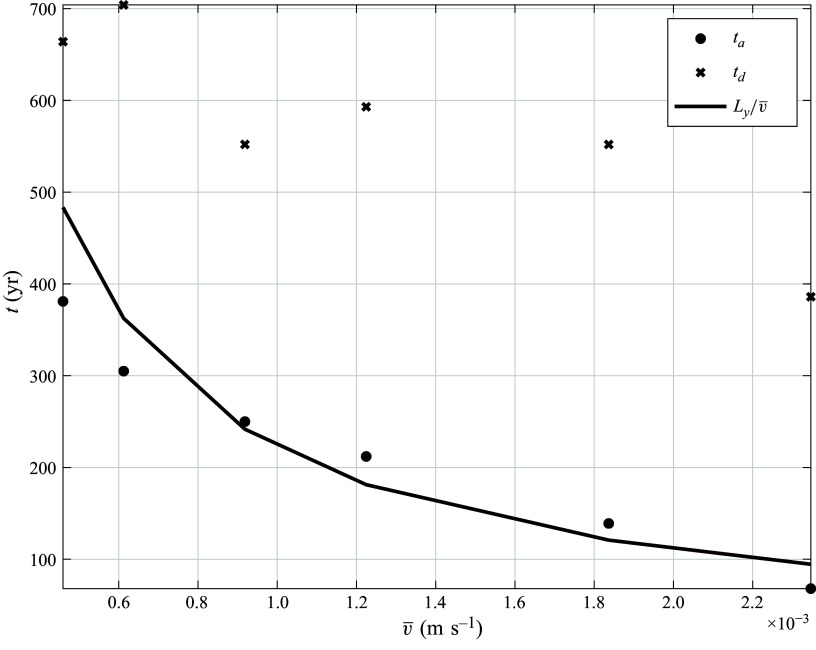
The advective time, 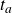 ( 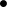 symbol), and diffusive time, 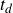 ( 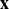 symbol), that best fit the invGauss FPTD in (1.1) as a function of the zonally average meridional velocity 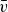 . Only values of 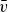 with 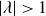 are considered. The solid curve is the theoretical prediction based on 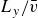 . The diffusive time associated with the stochastic noise alone would be 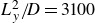 years.

In the range of parameters 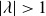 , where there are no direct exit paths, all parcels go through the gyres and the FPTD is well represented by the inverse Gaussian in (1.1). We expect the advection time, 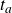 , to be determined by the zonally averaged throughflow, i.e. the gyres act to homogenise across the basin the net meridional transport associated with the boundary current. In this case, the theoretical estimate is 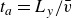 , where 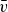 is the zonally averaged meridional velocity. Figure 10 compares this prediction (solid line) with the estimates best fitting the FPTD from the parcel simulations (filled circles). The prediction using the homogenised velocity is qualitative, but there are quantitative discrepancies, presumably because the homogenisation is not complete and not uniform in 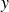 . In these simulations all parameters are held fixed, while 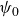 is varied. Also shown are the best fitted 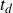 , which vary little as 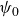 varies, indicating that the effective macroscopic diffusion depends on the stochastic noise and the gyral strength only.

## 5. The Levy distribution for flow in a `leaky’ pipe

As a model of the behaviour observed in the SOSE (figures 1 *b* and 3 *b*), and in the intermediate complexity models of § 4.1, we construct the FPTD for parcels advected by a narrow meridional boundary current and diffused across the current. Inspired by the `hairy-pipe’ model of Young (1988), we consider a two-dimensional problem in the 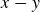 plane such that north–south advection ( 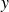 direction) is confined to a narrow region coupled with continuous diffusive `leakage’ out of the boundary current in the east–west ( 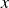 ) direction. In the following, we show that this configuration results in FPTDs of the Levy form (1.3).

The probability distribution, 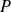 , of parcels at a point 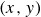 and time 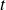 is governed by
(5.1)

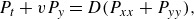

where 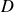 is the diffusivity and
(5.2)

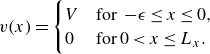

The velocity is a top hat confined to the narrow region 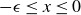 , which represents the width of the boundary current, and constant within this region. Considering the domain to be 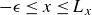 and 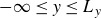 , the initial condition is
(5.3)

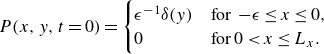

This flux-weighted release ensures the equivalence of Lagrangian parcels and tracer concentration distribution and is applied in the Lagrangian computations summarised in § 4 (Yang & Cessi 2024, Appendix A). Additional boundary conditions are 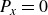 at 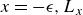 and 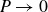 at 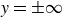 . The goal is to find the FPTDs for parcels (or tracer) starting at 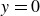 and arriving at 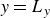 .

Within the narrow boundary layer, 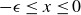 , we can consider the concentration of parcels to be well mixed in the 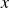 direction, while being advected by the constant meridional velocity 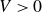 . Thus, for 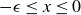 , 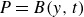 . Because the boundary layer is narrow, 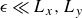 , we can also neglect diffusion in the 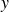 -direction. We can determine the evolution equation for 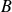 by integrating (5.1) in 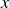 over 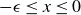 to find that
(5.4)

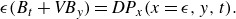

The assumption of a well-mixed concentration in the boundary layer holds as long as the advection is slow compared with the diffusion, i.e. 
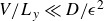
 .

For 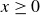 , there is no advection and just diffusion of tracer. Anticipating a solution that decays rapidly away from 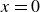 , we neglect diffusion in the 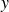 direction and 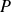 satisfies
(5.5)

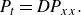

Despite the zero initial condition in the region 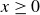 , there is a concentration 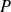 introduced by the diffusive flux leaking out of the boundary layer, i.e.
(5.6)

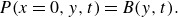

Integrating (5.5) in 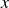 for 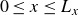 we find that 
(5.7)

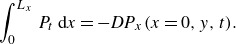

Summing (5.4) and (5.7) we obtain the evolution of the integral of 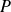 over the entire 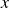 domain, governed by
(5.8)

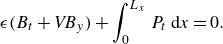

Because we neglect diffusion in the 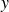 direction, and given the initial condition localised at 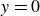 , no parcels are expected to be in the 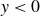 part of the domain (assuming that 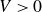 ). Therefore, 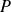 and 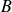 vanish for 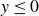 . The distribution of first passage time (FPTD) at the generic point 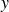 starting from the initial release at 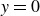 is given by (minus) the change in the survival probability (Cox & Miller 1965, Chapters 5.4, 5.7), i.e.
(5.9)

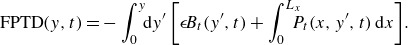

The right-hand side of (5.9) can be obtained by integrating (5.8) in 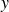 , and using the boundary condition 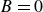 at 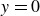 , to obtain
(5.10)

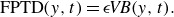

In order to find 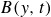 , we need to solve (5.4) in the region 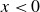 with 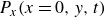 determined by solving the interior diffusion (5.5) for 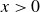 . The solution for the FPTD, denoted with `Levy1S’, can be found using the Laplace transform in the limit 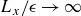 (Appendix B) and is given by
(5.11)

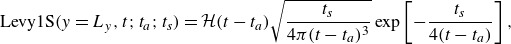

where 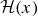 is the Heaviside function. This is the same expression as (1.3), with explicit expressions for the two time scales as a function of the physical parameters of the model. The advective time, 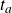 , and the scale parameter, 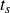 , are defined as
(5.12)

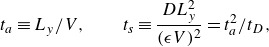

where 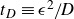 is the diffusive time across the boundary current. Because diffusion in the 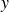 direction is neglected, no parcel can exit the domain before the advective time 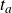 . After this time, the distribution changes on the time scale 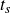 . This expression for the FPTD is a (one-sided) Levy distribution, with the shift parameter given by 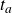 and the scale parameter given by 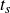 . Because of the slow 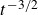 decay of Levy1S for large times, all moments of the distribution diverge, so we cannot calculate the average first passage time. For times 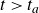 , (5.11) coincides with the FPTD of Brownian motion without drift, as appropriate in the limit 
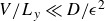
 .

Figure 6 shows that the distribution Levy1S (blue line) describes well the FPTD of parcels advected by a combination of stochastic noise plus a western boundary current that decays exponentially from the boundary, using 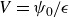 , i.e. the maximum velocity of the current. The fit (5.11) works well, even though 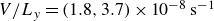 is not much smaller than 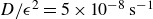 , indicating that this simplified model has a range of validity beyond the limit in which it was derived.

Figure 6 compares the numerically simulated distribution (light blue histogram) to the purely advective solution (4.4) (black dashed line) and the Levy’s distribution (5.11) (blue line). Even though the advective solution (4.4) uses the exact expression for the profile of the boundary current, it is a poorer agreement than the Levy’s distribution (5.11) using a constant velocity for the boundary current. This is because the parcels slowly leaking diffusively in the quiescent interior are important for the weakly decaying tail of the FTPD.

## 6. A two-peaked distribution with two diffusively coupled pipes

Motivated by the results in § 4.3, we further expand the two-dimensional model developed in § 5, by adding a weak meridional velocity outside the western boundary current region. This `outer’ meridional velocity represents the slow net meridional movement of parcels caught in the recirculating gyres. The rapid velocity in the western boundary current represents the fast meridional movement of parcels that can proceed without recirculating around the gyres. In the three-gyre model of § 4.3 these fast paths only exist for 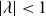 .

With these assumptions, the probability distribution, 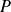 , of parcels at a point 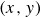 and time 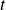 is governed by
(6.1)

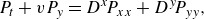

where
(6.2)

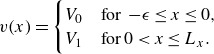

The domain is 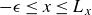 and 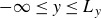 , where 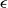 is the width of the fast throughflow western boundary current. We consider the diffusivity to be anistropic, because the tortuous paths induced by the gyres enhance the diffusivity in the 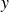 direction. To account for parcels that are deviated by the gyral circulation very close to the entry point at 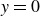 (cf. figure 8 *b*), we modify the initial conditions to
(6.3)

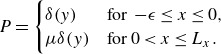

The parameter 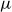 is the strength of the release applied in the large interior region. Considering that the gyral circulation deviates an 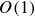 fraction of the parcels injected at 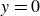 into the interior, we consider 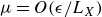 , so that the total transport in the boundary current and in the outer region are comparable. Additional boundary conditions are 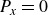 at 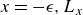 and 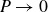 as 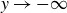 .

Within the narrow boundary layer, 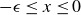 , the concentration of parcels is well mixed in the 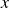 direction, while being advected by the constant velocity meridional velocity 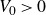 . Because the boundary layer is narrow, 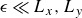 , we can also neglect diffusion in the 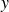 -direction. Thus, for 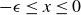 , 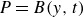 . We can determine the evolution equation for 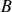 by integrating (5.1) in 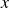 over the boundary layer region to find that 
(6.4)

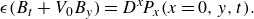




For 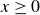 , there is weak advection and diffusion of tracer and 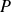 satisfies
(6.5)

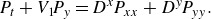

The concentration must be continuous at 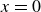 , so we have
(6.6)

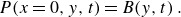

The details of the approximate solution can be found in Appendix C. The final expression for the FPTD at the point 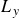 is
(6.7)

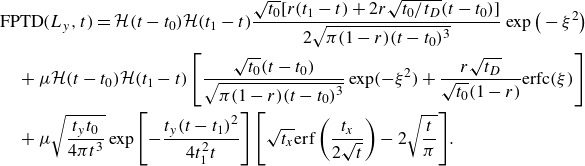




We have defined 
(6.8)

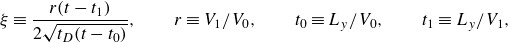



(6.9)

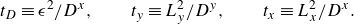




Here 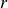 is the ratio of the meridional velocities, 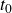 is the advective time along the fast boundary current and 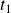 is the advective time in the slow interior region. These two advective time scales control the two peaks of the distribution. In (6.7), 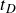 is the rapid diffusion across the boundary layer, 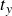 and 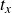 are the slow diffusive times along the full latitudinal and longitudinal extents of the domain, respectively. The diffusive time scales 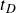 and 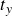 control the widths of the two peaks. The first term on the right-hand side of (6.7) is a modification to the expression for the LevyS1 (5.11) due the slow advection outside the boundary current, proportional to 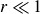 . The long time 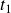 would allow the tail of the distribution to decay exponentially for large times. However, without meridional diffusion (neglected to obtain this term), all parcels exit the domain after a time 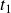 and none reach the absorbing boundary before the fast advection time 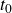 . Thus, the exponential decay for 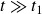 is not sampled by this term. The second term on the right-hand side of (6.7) accounts for the backscatter towards 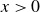 of parcels originally initialised in the interior then diffused back into 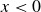 . The last term on the right-hand side of (6.7) is the inverse Gaussian arising from the advection by 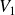 in the interior and the diffusion in the latitudinal direction, described by the parameter 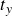 . Diffusion in the longitudinal direction, controlled by the parameter 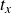 , plays a minor role: the contribution to the structure in the longitudinal direction is essentially constant in the parameter range of interest.

**Figure 11.**
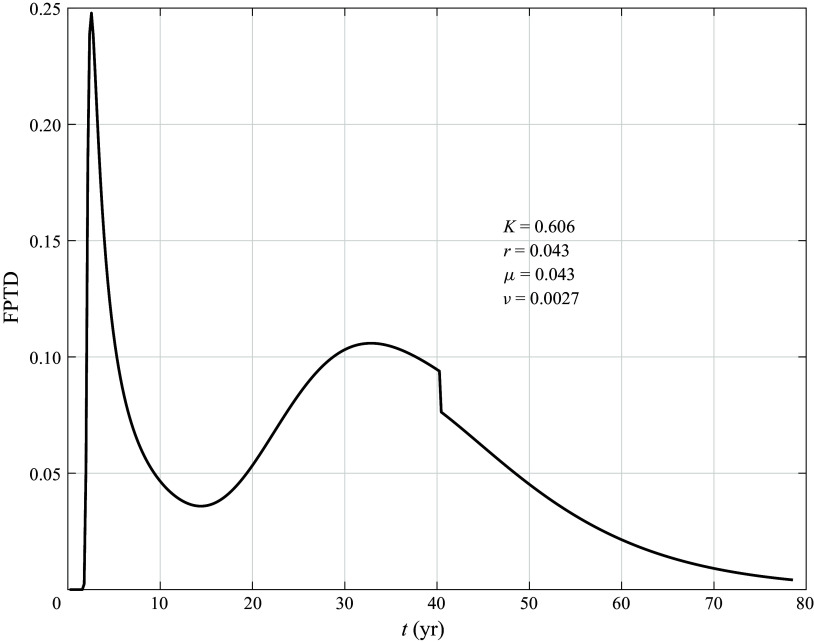
First passage time distribution (6.7). The values of the dimensional parameters are 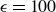 km, 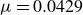 , 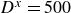 m 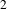 s^−1^, 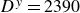 m 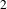 s^−1^, 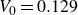 m s^−1^, 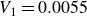 m s^−1^, 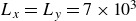 km. These values correspond to 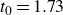 years, 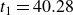 years, 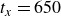 years, 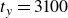 years. The values of the non-dimensional parameters introduced in Appendix C are reported in the legend.

An example of the FPTD for a reasonable choice of parameters is shown in figure 11. We do not attempt a detailed fit to the FPTD obtained by direct numerical integrations of the stochastic system (4.1). The important point is that there are two peaks in the distribution: the peak at short times, similar to the Levy1S distribution, is associated with the advection along streamlines in the western boundary current that do not recirculate in the interior gyres, e.g. the trajectory with filled circles in figure 8 *a*. The peak at later times is associated with the streamlines that recirculate around gyres and are advected by a slower meridional velocity distributed across the whole domain, while experiencing enhanced diffusion in the 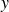 -direction associated with the stirring effect of the large-scale gyres. The analysis in § 4.3 indicates that the gyres increase the effective meridional diffusivity by a factor larger than one (but less than ten). In figure 11, 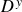 is increased by a factor of about five relative to 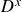 to illustrate this process.

The distribution exhibits two peaks: one at 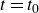 , associated with the first term on the right-hand side of (6.7), and one at 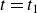 , associated with the inverse Gaussian on the third line of (6.7). The discontinuity at 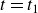 is associated with the corresponding Heaviside function in the FTPD in (6.7), and would be smoothed out by the addition of diffusion in the 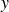 direction in the boundary layer region. When all the streamlines recirculate in the gyres, the width of the boundary region vanishes and we can set 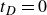 . Then, the only term that survives is the term on the third line of (6.7) proportional to the inverse Gaussian distribution.

## 7. Discussion

The different elements of the ocean general circulation, interhemispheric boundary currents, basin-scale gyres and mesoscale eddies, each contribute to a spectrum of transit times between target regions on the planetary scales. A subset of the possible rich behaviour encountered in the oceanographic context of multiscale flows is illustrated by the transit-time distributions of two disparate regions of the world ocean: the South Atlantic Ocean and the Western Mediterranean Sea.

Because of the connection with the Southern Ocean, the subtropical gyre of the southern hemisphere does not close within the Atlantic sector. Thus, upper ocean paths from the tip of South Africa to the equatorial region of the western South Atlantic are direct except for the dispersion effected by mesoscale eddies. This type of circulation is well described by a boundary current with noise. This configuration leads to FPTDs that lack exponential tails and decay with slow power laws for large times. This behaviour is in contrast with the classical paradigm of Brownian motion with constant drift, described by the inverse Gaussian, which decays exponentially for large times and has well-defined moments of the distribution.

In contrast, the Western Mediterranean Sea is connected to adjacent seas by narrow and shallow straits, which promote gyral circulations on several scales, in addition to boundary currents connecting the interior flow to the straits, especially to the Strait of Gibraltar. The net result of the interaction between these throughflow currents, the gyres and the eddies are transit-time distributions that, when integrated over the directions perpendicular to the throughflow are well described by a one-dimensional inverse Gaussian distribution.

In general, 3-D gyral circulation leads to transit-time distributions potentially quite complex, depending on the strength of the gyres relative to the throughflow currents and the mesoscale eddies. Insofar as eddies can be represented with white noise, some physical intuition can be obtained by considering Lagrangian parcels advected by steady 3-D flows plus stochastic noise. With weak or no stochastic noise, multimodal distributions are the norm, even when the origin of the parcels (or tracer) is localised to a small region. This is because the tortuosity of the paths varies among trajectories that start close together: parcels that are very close at the initial time, quickly diverge taking disparate times to cross the domain, even in the ensemble average. A fast throughflow boundary current further complicates the distribution, by adding quick paths that avoid the spiralling gyral routes.

When stochastic noise is added to throughflow boundary current plus gyres, the distribution can be unimodal or multimodal depending on the value of the parameters controlling the total velocity of the boundary current. If the boundary current changes sign along the boundary then all parcel paths recirculate within the gyres. Gyral recirculation increases the transit times by homogenising the net throughflow velocity over a broader region. At the same time, gyres increase parcel dispersion well above the typical values associated with mesoscale eddies. The net result is a FPTD that is broad and decays exponentially for large times.

## Data Availability

SOSE outputs are freely available from http://sose.ucsd.edu. Ariane version 2.3.0, is available at http://ariane.lagrangian.free.fr/ariane.html. For reproducibility of all major results of the Lagrangian analyses performed for this study, the customised code of the Ariane software, the simulated Lagrangian trajectories and post-processing scripts are made available through https://github.com/giuli9/Ariane-postprocessing and https://doi.org/10.5281/zenodo.7705093.

## Appendix A. The FPTD with a boundary current and no diffusion

Here we derive the FPTD for the western boundary current in the limit where  , i.e. when diffusion can be neglected everywhere. We consider a two-dimensional problem in the  plane such that north–south advection decays exponentially away from  . In this case the probability distribution,  , of parcels at a point  is governed by

(A1)

with  , in the domain  ,  . The initial condition is flux weighted with the meridional velocity, i.e.

(A2)

The solution for  is

(A3)

Integrating in  and  to find the survival probability and then taking minus its derivative (Cox & Miller 1965, Chapters 5.4, 5.7), the FPTD at  is given by

(A4)

where  .

## Appendix B. A ‘leaky’ pipe with constant velocity

From Laplace transforming (5.5) we have

(B1)

subject to the boundary conditions  as  and  at  . The solution, neglecting terms that decay exponentially for  , is

(B2)

In the region  , taking the Laplace transform of (5.4),  obeys

(B3)

where the right-hand side has been evaluated using (B2). We can solve (B3), subject to the initial condition  , and the boundary condition  at  to find that

(B4)

Using tables of inverse Laplace transforms we find that (Abramowitz & Stegun 1968)

(B5)

where  is the Heaviside function. Using the relation (5.9) and evaluating the FPTD at the exit point  , we find that

(B6)

Even though there is no throughflow velocity in the region  , the concentration of parcels  does not vanish at  , because it is diffused from the boundary layer region, and thus, decays rapidly away from  .

The solution (B6) shows that there are two time scales: the fastest advective time,  , and the scale parameter,  , where  is the diffusive time across the boundary current. No parcel can exit the domain before the fastest advective time  , but, after this time, the distribution changes on the time scale  . The distribution (B6) is the (one-sided) Levy distribution.

For completeness, the expression for  is given by

(B7)

## Appendix C. Two diffusively coupled pipes

The solution for the FPTD problem formulated in § 6 is best approached in non-dimensional form. We use the following scales:

(C1)

In terms of the non-dimensional variables, denoted by a hat, the governing equations become

(C2)

(C3)

where  is the Peclet number in the latitudinal direction and  is the non-dimensional width of the western boundary layer.

We can solve (6.5) for  for  by transforming to a new set of coordinates defined as

(C4)

so (6.5) becomes

(C5)

We can separate the solution in two parts,  . Here  is associated with the forcing in the boundary current region,  , while  is due to the forcing in the interior region,  , proportional to  . The procedure to determine  closely follows the method used in Appendix B. Taking the Laplace transform of (C5) we find that

(C6)

Anticipating a solution that decays away from  on a scale of  , and for  , we can neglect diffusion in the  direction, and we find that

(C7)

The solution for  is given by

(C8)

The solution (C8) satisfies (C5), it vanishes at  ,  and  , i.e. the location of the absorbing boundary. Here  has zero derivative as  and becomes  at  . There is a weak diffusive backscatter for  , but  as  . In the  plane,  follows the classical solution of advection-diffusion in one dimension of tracer concentration released impulsively at  and absorbed at  (Cox & Miller 1965, Chapter 5.7). Here, there is a weak modulation in  , on a scale of  , because of the requirement that  at  and  at  .

The response induced by  in the region  is not amenable to explicit integration. However, the solution for  can be obtained using the limit  of  , which is a decent approximation given the smallness of  . In this limit  becomes

(C9)

Using this approximate form, we are now in a position to solve (5.4) in the narrow boundary region,  . Using the transformed variables  and taking the Laplace transform in  of (C3), we have

(C10)

Because we are neglecting diffusion in the  direction, and given that the initial condition is localised at  ,  for  . The solution for the Laplace transform  that satisfies all the boundary conditions is

(C11)

The Heaviside term  arises because the  function initial condition is picked up only for  . For  , we pick up the homogeneous boundary condition at  and the solution vanishes. This is not surprising since  occurs for times  , i.e. longer than the advection time in the outer region where the velocity is slow. After this long time, and without meridional diffusion, all parcels have gone past the latitude  , leaving nothing behind. Taking the inverse Laplace transformation and going back to the original  coordinates  becomes

(C12)

Using (C7), the solution for  is

(C13)

The FPTD can be obtained by combining the contributions from the two regions, weighted by their widths in the  direction. In other words, we integrate (C3) for  and (C2) for  , sum the resulting integrals and further integrate in  to obtain

(C14)

where  is the non-dimensional width of the outer region. Using the solutions for  ,  and  in (C8), the FPTD at the absorbing point,  , is given by

(C15)

where  is given in (C12). The dimensional version of this result is given in (6.7).
